# Systemic juvenile idiopathic arthritis in French Afro-Caribbean children, a retrospective cohort study

**DOI:** 10.1186/s12969-022-00766-8

**Published:** 2022-11-16

**Authors:** A. Felix, F. Delion, B. Suzon, S. Pallara-Sirven, N. Elenga, P. Quartier, F. Louis-Sidney, M. Dramé, Y. Hatchuel

**Affiliations:** 1 Department of Pediatrics, Martinique University Hospital, Fort-de-France, France; 2 MFME. CHU de La Martinique La Meynard, 97261 Fort-de-France, France; 3 Department of Pediatrics, Guadeloupe University Hospital, Pointe-À-Pitre, France; 4 Department of Internal Medicine, Martinique University Hospital, Fort-de-France, France; 5 Department of Pediatrics, Andrée Rosemon Hospital, Cayenne, France; 6 Department of Pediatric Rheumatology, Necker Hospital, Paris, France; 7 Department of Rheumatology, Martinique University Hospital, Fort-de-France, France; 8 Department of Clinical Research and Innovation, Martinique University Hospital, Fort-de-France, France

**Keywords:** Systemic juvenile idiopathic arthritis, Still disease, Macrophage activation syndrome, Afro-Caribbean children

## Abstract

**Introduction:**

The epidemiology and clinical presentation of systemic juvenile idiopathic arthritis (sJIA) in the Afro-Caribbean population is not well described.

**Methods:**

Retrospective study conducted between January 2000 and January 2022 in the French Overseas Departments of America. Clinical data were obtained from multiple sources: computerized hospital archives, registries of referring pediatricians, and the French National Registry for rare diseases. The disease studied was sJIA defined according to international criteria.

**Results:**

Twenty-five patients were identified. Mean age at diagnosis was 7.5 years (range: 1.2—14.9 years) and mean duration of follow-up was 5.2 years (range: 0.5—16 years). All patients had joint involvement at diagnosis with 68% presenting inflammatory arthritis and 32% inflammatory joint pain. Sixteen percent had coronary involvement at onset. More than half (52%) suffered from macrophage activation syndrome (MAS) during childhood (32% at onset). The mean number of flares in childhood was 2 (Range: 1—5). Sixty-eight percent of patients had disease control during childhood without biotherapy. The most frequent second line treatment was anakinra (7/8). There was no difference in clinical or biological severity according to gender. The median duration of treatment during childhood was 5 months (range: 2—144) and 72% had a cumulative treatment duration of less than one year.

**Conclusion:**

These patients of Afro-Caribbean origin suffering from sJIA showed some specificities, such as a higher rate of MAS and coronary involvement at onset. The incidence per year was stable over a 20-year period. Overall outcomes during childhood were similar to western countries.

## Key messages

1. First cohort of pediatric patients with sJIA of Afro-Caribbean origin
2. Higher rate of coronary involvement at onset
3. Highest rate of macrophage activation syndrome during childhood described
4. Incidence and overall prognosis similar to Western countries

## Introduction

In populations of African descent, the clinical presentation and epidemiology of auto-immune and auto-inflammatory diseases is quite particular [1, 2]. The epidemiology of Still’s disease, or systemic juvenile idiopathic arthritis (sJIA), in the Afro-Caribbean population has not been well described heretofore. The French overseas departments of America (FOAD, i.e. Martinique, Guadeloupe, and French Guyana) have a combined population of approximately 330,000 children aged under 18 years. The health system there is free and universal, with 2 university hospitals and a regional competence center approved by the French Ministry of Health. Although there are no official ethnic statistics, because it is prohibited by French law to collect data on ethnic origin, a large majority of patients are of black African descent [3] (> 90%). The objectives of this study were to perform a retrospective, descriptive study, reported according to the STROBE methodology [4], of patients from the FOAD followed for sJIA between 2000 and January 2022. We aimed to describe their clinical, biological specificities and outcomes during childhood.

## Methods

This was a retrospective study conducted over the period from January 2000 to January 2022. We included children aged 0 to 16 years at diagnosis of the disease. The methodology for patient identification aimed to cross-reference different sources to ensure exhaustive identification of all patients. In each referenced center of the FOAD, we searched the local registries of pediatric patients followed for JIA and auto-inflammatory syndrome by the referring pediatricians. We extracted lists of patients from the electronic hospital archives using the coded diagnosis of JIA and auto-inflammatory syndromes from 2000 to 2022. We also extracted the list of patients recorded with JIA and auto-inflammatory syndrome in the electronic French national registry for rare diseases (BAMARA), a secure national information system that gathers clinical data of patients affected by rare diseases in a data warehouse. Subsequently, the lists of patients were analyzed for the relevance of the diagnoses, to check inclusion criteria and to eliminate duplicates. Data were retrieved by consulting follow-up reports and hospitalization reports available in the patients’ medical files. The disease studied was sJIA of pediatric onset according to international ILAR criteria [5, 6]. Macrophage activation syndrome (MAS) complicating sJIA was also defined according to international guidelines [7]. For patients treated before the publication of the new criteria, we retrospectively checked the medical records to see whether they met the criteria for sJIA and/or MAS complicating sJIA. We defined coronary dilatation as a diameter > 2 standard deviations for body surface area. Mucosal involvement was defined as erythema, edema, aphthous ulcers, or ulceration in the nose and throat area. A clinical flare was described as a clinical and laboratory manifestation requiring a change in background therapy or glucocorticoid pulse (> 0.5 mg/kg/day). The treatment of a flare was protocolized, with a loading dose of 1-2 mg/kg/day. In the event of a good clinical response to glucocorticoids, they were gradually tapered and weaned over 3–4 months.

Patients were included if they (or their parents) were born, raised, and resident in the FOAD, while those present for a short stay (vacation) were excluded.

Data are described as means, medians, and range for quantitative variables, or as number and percentage for categorical variables. Differences in percentages were tested with the chi square test or Fisher’s exact test for expected frequencies < 5, using STATA software. A *p*-value < 0.05 was considered statistically significant. The Institutional Review Board of the University Hospital of Martinique approved the study under the number 2021/116.

## Results

**Fig. 1:**
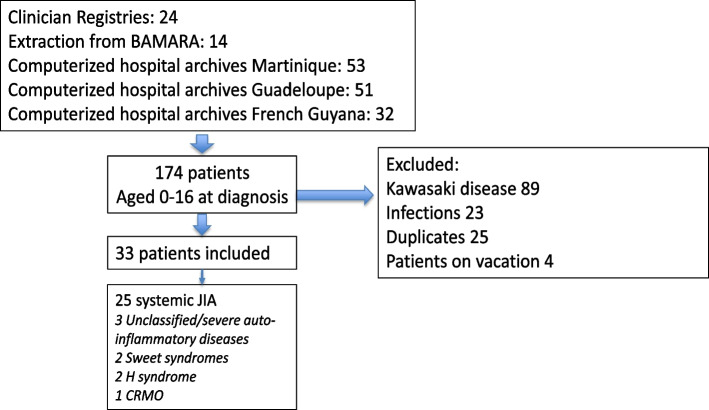
Flowchart of the study population. BAMARA is the French National registry for rare disease. H syndrome: Histiocytosis lymphadenopathy plus syndrome. CRMO: Chronic recurrent multifocal osteomyelitis

**Fig. 2:**
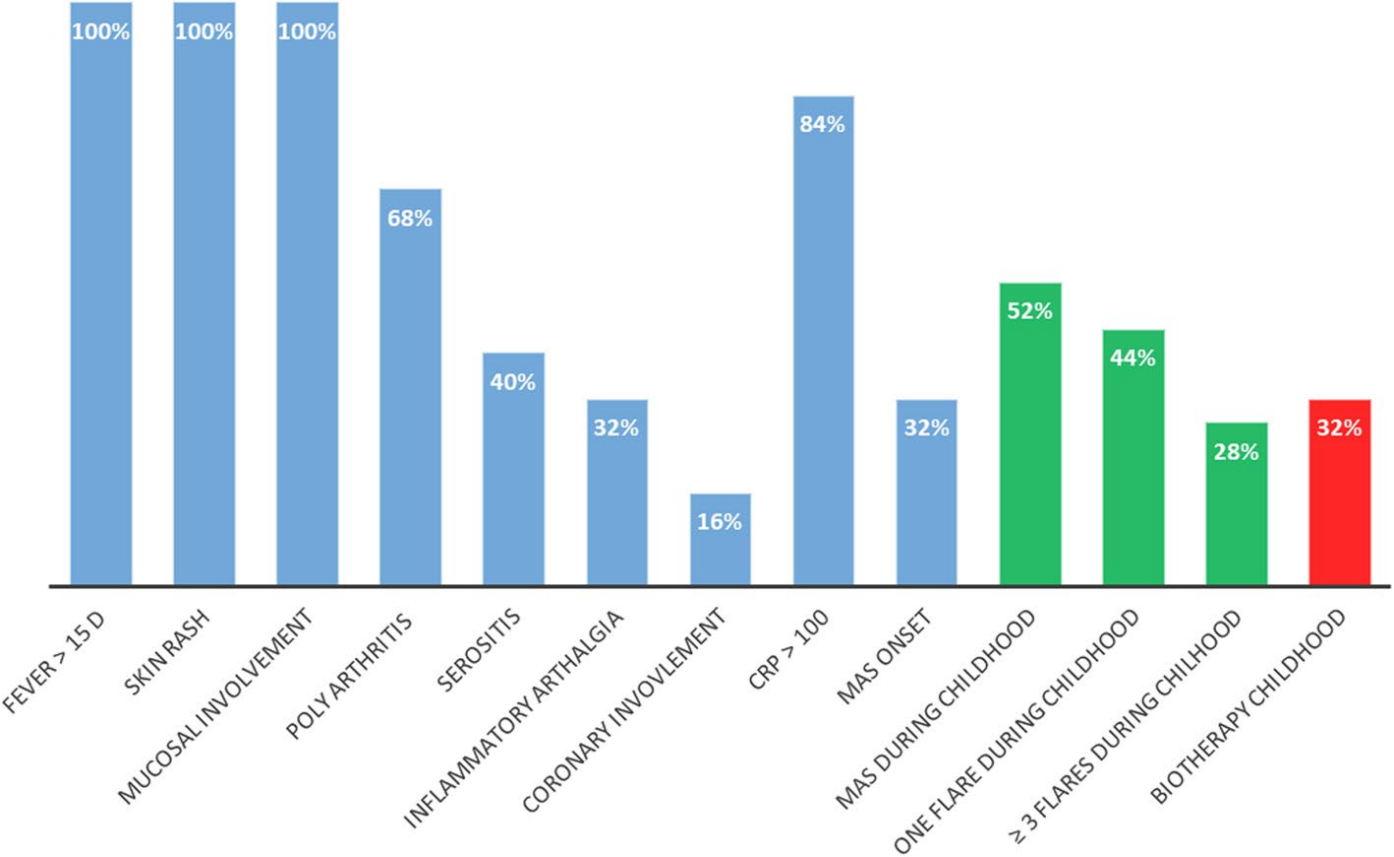
Clinical and biological characteristics of our cohort of sJIA. Mucosal involvement was defined as oral erythema, edema, aphthous ulcers, or ulceration in the nose and throat area. MAS: Macrophage activation syndrome. Green bars show cumulative data during childhood. A patient with known or suspected sJIA was considered as having MAS if Ferritin > 684 ng/ml and 2 of the following criteria were met: Platelet count ≤ 181 × 109/liter, Triglycerides > 156 mg/dl, Fibrinogen ≤ 360 mg/dl, Aspartate aminotransferase > 48 units/liter. Every patient in our cohort had at least 4/5 criteria

One hundred and seventy seven patients were identified through the registries over the 22 year period; of whom 33 were identified as having auto-inflammatory syndrome, including 25 patients with sJIA (Fig. 1). The sex ratio was 2/3 boys and 1/3 girls. The mean age at diagnosis was 7.5 years (range: 1.2—14.9 years). There was no notable age distribution (40% under 5 years old, 32% between 5 and 10 years old and 28% between 10 and 15 years old). The median time from symptom onset to diagnosis was 2 months (range: 0.1—12 months). The mean duration of follow-up was 5.2 years (range: 0.5—16 years). The clinical and biological characteristics of the study population are shown in Fig. 2 and Table 1. All patients had joint involvement at diagnosis, with 17 presenting polyarthritis (68%) and 8 presenting inflammatory joint pain, with painful limitation of movement (32%). Ten patients (40%) had serositis (7/10 pericarditis and 3/10 pleuritis). Four patients had coronary involvement at diagnosis (16%); they all had coronary dilatation (from 3.4 to 5.2 mm) without aneurysm and without further complications, such as later coronary artery damage or recurrence (Table 1). All patients with coronary dilatation had a favorable outcome, and all dilation had disappeared at the control echocardiography (2 months later).

**Table 1:**
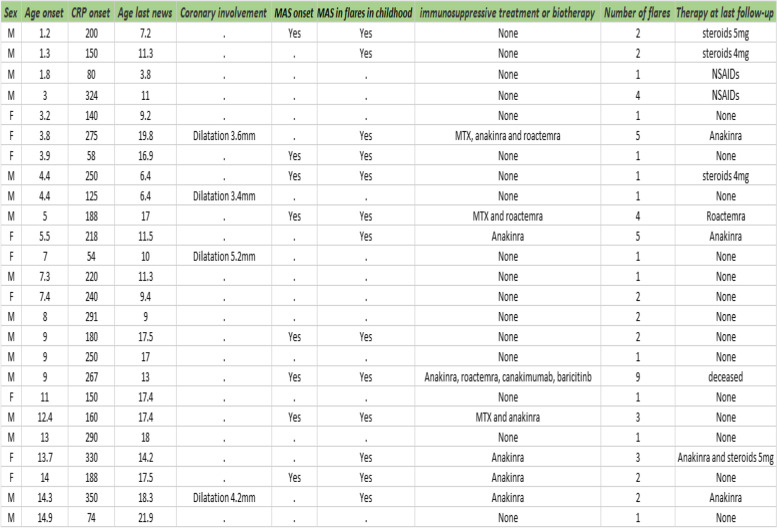
Description of patients with sJIA

Time to diagnosis is in months, follow-up is in years, MAS stands for Macrophage activation syndrome. Age is in years, CRP is in mg/ml. 0 stands for no treatment

No patient had uveitis during follow up. Only one patient with a severe phenotype, including severe flares with MAS refractory to biotherapies, suffered from lung disease associated with sJIA and developed pulmonary hypertension. The average CRP at diagnosis was 180 mg/L. Only 3/22 had positive anti-nuclear antibodies and none had a positive rheumatoid factor or Anti-citrullinated protein antibodies. We found a viral infectious trigger (considered as positive nasal or blood PCR or serology showing IgM antibody) in 15/25 patients (60%). At onset, every patient had a complete bacteriological assessment including several blood cultures, all of which were negative.

Eight patients had MAS at diagnosis, and five developed MAS during flares in childhood according to the international classification [7] (13/25, 52% of patients during childhood). They all presented organ enlargement and biological abnormalities (4/5 criteria). Eight of these patients (62%) required the addition of biotherapy to control a flare of MAS after failure of glucocorticoid therapy (anakinra in second line 7/8). Three of them (23%) required two different biotherapies for severe MAS (anakinra and tocilizumab). The mean and median number of flares in childhood was 2 per patient (1—5) (including the initial flare). Seventeen patients had a disease control during childhood with glucocorticoids and non-steroidal anti-inflammatory drugs (NSAIDs) (68%). Eleven patients (44%) had only one flare of sJIA during childhood. Eight patients (32%) required a second therapeutic line for MAS control or glucocorticoid-sparing. Most of them (7/8) suffered from at least 3 flares during childhood and had MAS associated with sJIA at some point during their disease course. No patient in our cohort without MAS during childhood required biotherapy as background therapy. There was no difference in clinical and biological features according to gender (for boys versus girls respectively: MAS during childhood 50% vs 55% (*p* = 0.8), average number of flares during childhood 2.31 vs 2.33 and second line with biotherapy 25% vs 44% (*p* = 0.39)).

We calculated the cumulative duration of treatment for our patients; the median was 5 months (range: 2—144) and 72% had a cumulative treatment duration of less than one year during childhood. With a median follow-up of 5.2 years and median cumulative treatment duration of 5 months, our patients were *on therapy* (taking any medication) only 8% of the time. Based on the latest data available, 21/25 patients were successfully weaned off glucocorticoids during childhood (84%). All unweaned patients were still on glucocorticoid therapy at less than 5 mg per day, or NSAIDs. Twelve patients (48%) transitioned to adult care for continued follow-up; all but one had no background treatment for a year. There was one fatal severe infectious event, namely one patient with pulmonary hypertension died from COVID-19 infection.

## Discussion

This retrospective study from the FOAD over a period of 22 years identified a cohort of 25 patients suffering from sJIA. This is the largest cohort of pediatric patients with this disease of Afro-Caribbean origin. The current international JIA classification has contributed to a more uniform classification of this disease, thus facilitating comparisons of diagnosis and epidemiology across countries and ethnic populations [5]. One of the strengths of this work is its multicenter nature, with the participation of all the referring pediatricians in the FOAD. Our methodology enabled us to analyze patient therapies by referring to the national registry for rare diseases, as well the registries of local referring clinicians and by exploring computerized hospital archives with wide inclusion criteria. This led to exhaustive identification of patients, and we cross-checked data from multiple sources to minimize the loss of patients and data. Thus, the prevalence in our study may have been underestimated, because of the memory bias of retrospective studies covering a long period. There is also potential for recruitment bias in our study, because our methodology mostly identifies patients who required hospitalization. Nevertheless, most patients with sJIA have a short initial hospitalization at disease onset.

Our study shows a stable incidence of sJIA in this population of Afro-Caribbean children over a 22-year period, at a rate of 0.4 patients per year per 100,000 children. This incidence is consistent with that described in Western countries [8, 9]. However, this contrasts with the rates of three main systemic diseases in the FOAD (namely systemic lupus, bowel disease and type 1 diabetes) with pediatric onset showing an increase in the number of cases over the years (unpublished personal data). The overall prognosis of the children in our cohort was similar to that of cohorts from Western countries [9, 10]. In the biotherapy era, with all biotherapies available in 2009 in the FOAD, more than two-thirds of children did not require biotherapy to achieve complete remission during childhood. None of our patients had ophthalmological involvement, which is consistent with the low incidence of uveitis in this subtype of JIA [11]. The proportion of patients with coronary involvement at onset was higher in our cohort. Coronary damage has previously been described in sJIA [12], but none of our patients had subsequent complications, such as later coronary artery damage, dilatation or relapse. The diagnosis of sJIA was made in the presence of evidence of persistent arthritis and/or MAS, and these patients did not have a more severe clinical or biological course. Our patients had few autoantibodies, which clearly differentiates this entity from other JIA or systemic disease [13]. Although we chose an international definition of MAS associated with sJIA [7] to avoid it simply reflecting the perception of the clinicians, we found a high percentage of MAS in our cohort, higher than previously described [14], which was not related to an increase in mortality. However, in our cohort most of the children who had MAS at some point during the course of their diseases required biotherapy for disease control. MAS complicating sJIA occurs more frequently during childhood than in adult onset Still’s disease [15]. Our patients were on therapy for a small part of the time they were being followed up by specialists, and a large majority of those who transitioned to adult care had been weaned from treatment for over a year. This probably illustrates the pediatricians’ doubts about the future outcomes of these patients [16]. Adult/child studies on the evolutionary profile of these patients are necessary to assess the risk of recurrence in adulthood.

## Conclusion

To the best of our knowledge, this cohort of patients followed in the FOAD is the largest focusing on children of Afro-Caribbean origin treated for sJIA in a developed-country health system. It describes their clinical and biological specificities, such as a higher rate of MAS and coronary involvement at onset. The incidence per year was stable over a 20-year period. Their overall outcomes during childhood were similar to those reported in western countries.

## Data Availability

All data generated or analyzed during this study are included in this published article and tables.

## Abbreviations

sJIA: Systemic juvenile idiopathic arthritis
FOAD: French overseas departments of America
MAS: Macrophage activation syndrome
NSAIDS: Non-steroidal anti-inflammatory drugs
